# Analysis of the R1b-DF27 haplogroup shows that a large fraction of Iberian Y-chromosome lineages originated recently in situ

**DOI:** 10.1038/s41598-017-07710-x

**Published:** 2017-08-04

**Authors:** Neus Solé-Morata, Patricia Villaescusa, Carla García-Fernández, Neus Font-Porterias, María José Illescas, Laura Valverde, Francesca Tassi, Silvia Ghirotto, Claude Férec, Karen Rouault, Susana Jiménez-Moreno, Begoña Martínez-Jarreta, Maria Fátima Pinheiro, María T. Zarrabeitia, Ángel Carracedo, Marian M. de Pancorbo, Francesc Calafell

**Affiliations:** 1 Institut de Biologia Evolutiva (CSIC-UPF), Departament de Ciències Experimentals i de la Salut, Universitat Pompeu Fabra, Barcelona, Catalonia Spain; 2 BIOMICs Research Group, Lascaray Research Center, University of the Basque Country UPV/EHU, Vitoria-Gasteiz, Spain; 3 Dipartimento di Scienze della Vita e Biotecnologie, Università di Ferrara, Ferrara, Italy; 4 Inserm, UMR 1078 Brest, France; 5 Laboratoire de Génétique Moléculaire, CHRU Brest, Hôpital Morvan, Brest, France; 6 Université de Bretagne Occidentale, Brest, France; 7 Etablissement Français du Sang-Bretagne, Brest, France; 8 Forensic and Legal Medicine Area, Department of Pathology and Surgery, University Miguel Hernández, Elche, Spain; 9 Department of Forensic Medicine, University of Zaragoza, Zaragoza, Spain; 10 Forensic Genetics Department, National Institute of Legal Medicine and Forensic Sciences, Porto, Portugal; 11 Unit of Legal Medicine, University of Cantabria, Santander, Spain; 12 Genomic Medicine Group, CIBERER- University of Santiago de Compostela, Galician Foundation of Genomic Medicine (SERGAS), Santiago de Compostela, Spain; 13 Center of Excellence in Genomic Medicine Research, King Abdulaziz University, Jeddah, Saudi Arabia

## Abstract

Haplogroup R1b-M269 comprises most Western European Y chromosomes; of its main branches, R1b-DF27 is by far the least known, and it appears to be highly prevalent only in Iberia. We have genotyped 1072 R1b-DF27 chromosomes for six additional SNPs and 17 Y-STRs in population samples from Spain, Portugal and France in order to further characterize this lineage and, in particular, to ascertain the time and place where it originated, as well as its subsequent dynamics. We found that R1b-DF27 is present in frequencies ~40% in Iberian populations and up to 70% in Basques, but it drops quickly to 6–20% in France. Overall, the age of R1b-DF27 is estimated at ~4,200 years ago, at the transition between the Neolithic and the Bronze Age, when the Y chromosome landscape of W Europe was thoroughly remodeled. In spite of its high frequency in Basques, Y-STR internal diversity of R1b-DF27 is lower there, and results in more recent age estimates; NE Iberia is the most likely place of origin of DF27. Subhaplogroup frequencies within R1b-DF27 are geographically structured, and show domains that are reminiscent of the pre-Roman Celtic/Iberian division, or of the medieval Christian kingdoms.

## Introduction

Although it contains ~1% of the genome length in a human male cell, the lack of recombination along most of the Y chromosome makes constructing phylogenies for genetic variation relatively easy. Coupled with a robust geographic differentiation, this trait has provided a comprehensive phylogeography of Y chromosome haplotypes (usually referred to as haplogroups), that has been thoroughly characterized. Thus, the origin, dispersal, and geographic spread of many haplogroups are known. Moreover, both the genotyping of fast-mutating short tandem repeats (STRs) in the non-recombining region of the Y chromosome (NRY), and the recent availability of ascertainment-bias-free whole sequences of the NRY have reliably added a temporal scale to the deployment of the Y-chromosome diversity. One of the most salient features of the recent evolutionary history of human Y chromosomes is that it seems to have happened in bursts, with haplogroups rising to high frequency in the wake of major lifestyle shifts and technological innovations such as the advent of the Neolithic or the recently acknowledged demographic upheaval caused by the Bronze Age in Europe^1, 2^.

The most frequent Y-chromosome haplogroup in W Europe is R1b-M269, with frequencies ranging from 41% (Germany) to 83% (Ireland)^3^. Precisely, the higher frequency of this haplogroup in W Europe rather than in E Europe or W Asia led previous authors to believe it had a post-glacial Palaeolithic origin^4, 5^; however, a larger STR variance in SE European and W Asian R1b-M269 chromosomes and direct TMRCA dating pointed to R1b-M269 having surfed the Neolithic wave of advance^3, 6^, the evidence for which other authors did not find conclusive^7^. Finally, direct dating from NRY sequence variation puts the origin of R1b-M269 in the Early Bronze Age, ~4500 years ago (ya)^1, 8^, consistent with the growing ancient DNA record, where a surge in R1b-M269 is indeed seen at that time^2, 9^. Note, though, that R1b-M415, a branch ancestral to R1b-M269, was found as early as 14,000 ya in Italy^10^ and 7,000 ya in Spain^2^. Moreover, lack of structure of STR variation within R1b-M269^11, 12^ points also to an explosive growth.

**Figure 1:**
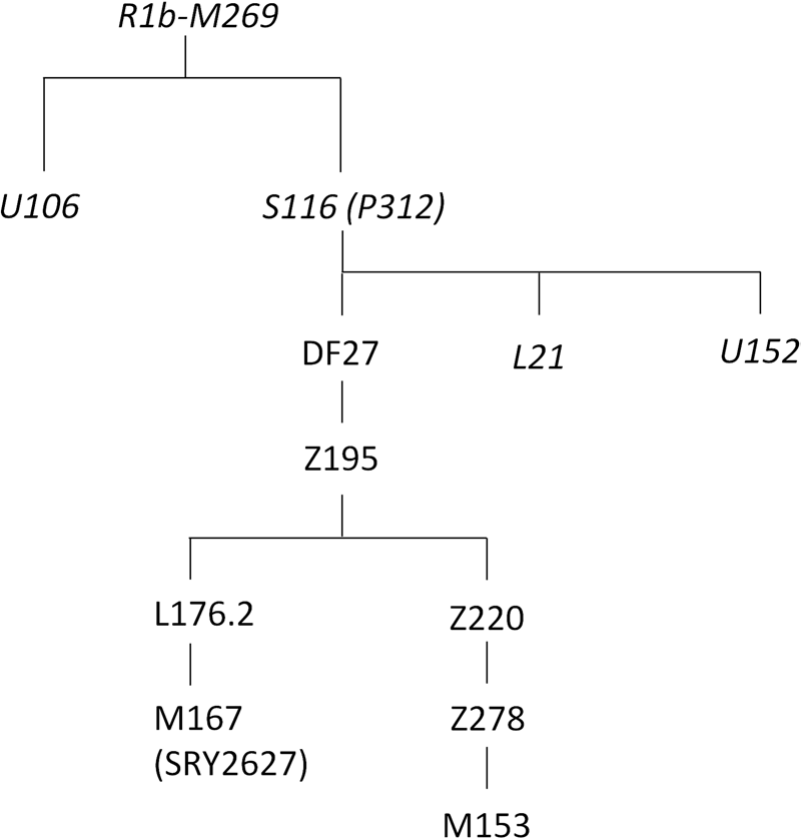
Simplified phylogenetic tree of the R1b-M269 haplogroup. SNPs in italics were not analyzed in this manuscript.

The most important branches of R1b-M269 are R1b-U106, particularly frequent in the Low Countries and NW Germany^3, 13^, and R1b-S116 (also known as R1b-P312), which is common throughout W Europe^3^. The latter trifurcates in turn into U152 (frequent in N Italy and Switzerland^13^), L21 (also known as M529, abundant in the British Isles^7^), and DF27 (Fig. 1; Supplementary Figure 1). DF27 was first discovered by citizen scientists^14^ and, although among the burgeoning amateur genetic genealogy it is known to be frequent in Iberian populations and their overseas offshoots, few academic publications have been devoted to it. It was found in the 1000 Genome Project populations at a frequency of 49% in Iberians, 6% in Tuscans, 7% in British, and it was absent elsewhere except for admixed populations in the Americas: Colombia (40%), Puerto Rico (36%), Mexico (10%), Perú (8%), African-Americans (4%) and Afro-Caribbeans (2%)^14, 15^. It was first genotyped specifically in a few Iberian populations, Brittany and Ireland as part of a study on R1b-S116^16^, which indeed confirmed that R1b-DF27 is present at frequencies >40% in Spain and Portugal. Subsequently, 12 SNPs within DF27 were genotyped in four N Spanish populations^17^, confirming its high frequency and hinting at some substructure within the Iberian Peninsula. As for its presence elsewhere, the frequency of R1b-S116 (xL21, U152) can be used as an upper bound for the frequency of R1b-DF27. R1b-S116 (x L21, U152) was found at frequencies 0–10% in Germany^3, 18^, 7% in the Netherlands^3^, 8–12% in Flanders^19^, 6–12% in Switzerland^3^, and 1–12% in Italy^3, 20^.

Here, by extending population sampling to cover France as well as by improving coverage in the Iberian Peninsula, we aim to i) further characterize the spatial distribution of R1b-DF27, ii) estimate its time of origin, and iii) explicitly model its expansion in relation both to its phylogenetic context, and to the demographic events that thoroughly reshaped the genetic diversity of W Europe around 4500 ya.

## Results

**Figure 2:**
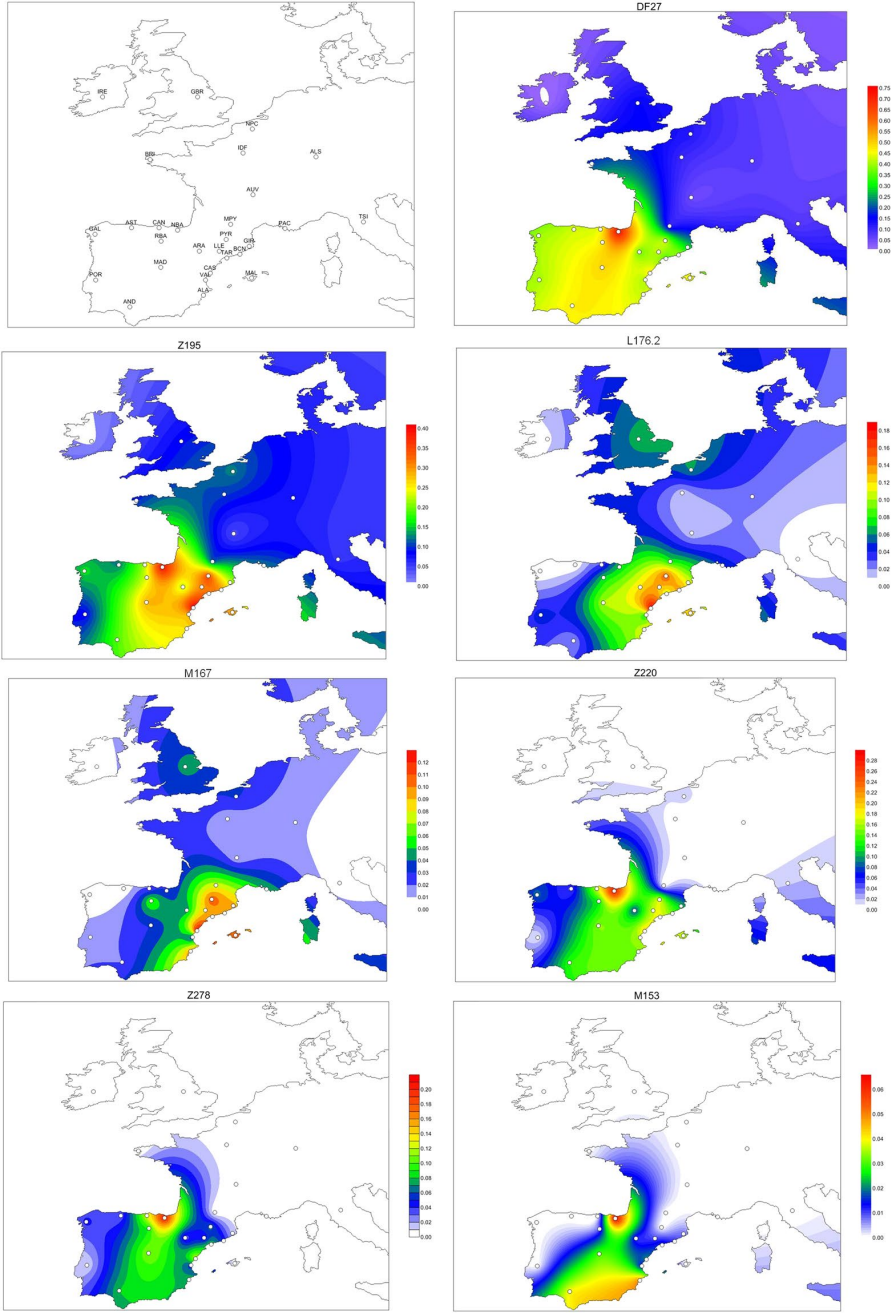
Contour maps of the derived allele frequencies of the SNPs analyzed in this manuscript. Population abbreviations as in Table 1. Maps were drawn with SURFER v. 12 (Golden Software, Golden CO, USA).

Over one thousand individuals carrying DF27 were typed for six additional SNPs (Table 1, Fig. 1) and 17 Y-STRs. DF27 itself was found at frequencies 0.3–0.5 in Iberia (with a mean of 0.42), with the notable exception of native Basques, where it reached 0.74 (for this and all subsequent frequency values, see Fig. 2 and Supplementary Table 1). In France, it dropped to a range of 0.06–0.20 and a mean of 0.11. Elsewhere, it was 0.15 in Britain (but <0.01 in Ireland) and 0.08 in Tuscany. Most (50–100%, with a proportion that dropped from East to West) DF27 Y chromosomes were also derived for Z195; thus, the highest frequencies of Z195 (0.29–0.41) were reached both in the Basque Country and in E Iberia (Catalonia, Valencia), and it becomes as rare in Portugal as it is in France. Conversely, the highest frequencies of R1b-DF27* (xZ195) are found in Native Basques and Western Iberian populations such as Asturias, Portugal and Galicia, which may harbor yet unknown branches of R1b-DF27. In turn, Z195 splits into two branches, namely L176.2 and Z220 (Fig. 1). Note that L176 is a recurrent mutation that defines two clades in the Y phylogeny: L176.1 within R1a, and L176.2 under R1b-DF27; throughout this manuscript, we will refer exclusively to the latter. L176.2 and Z220 peak, respectively, in E Iberia and the Basque Country. L176.2 is further subdivided into M167 (SRY2627, ref. 21), with the highest frequencies in Catalonia and the lands settled from Catalonia in the 13th century (Valencia, the Balearics). This marker had been typed in a number of Iberian and other European populations^4, 18–20, 22–25^, and the overall frequency pattern found (Supplementary Figure 1) confirms a distribution centred in the eastern half of Iberia, although with higher frequencies (up to 0.16) in the upper Ebro river valley and the Pyrenees. As mentioned above, Z220 is most frequent in the Basque Country (0.28), and a similar pattern is found for its successive nested clades, namely Z278 and M153. For the latter, available additional data^22, 23, 25^ showed it confined to the Iberian Peninsula, with frequencies 0.06–0.40 among Basque subpopulations, but rarely above 0.01 elsewhere (Supplementary Figure 1).

**Table 1:** Populations sampled and their original sources.

Population	abbr.	Region	lon	lat	Total N	Source	N DF27 typed	Additional R1b(1)	Additional P312 (2)	Additional Z195 (3)	SNP typing	STR typing
Alacant	ALA	Valencia	−0.56	38.36	142	(113)^16^, (29)^27^	22	0	0	0	this work^16, 27^	[27], [54]
Alsace	ALS	France	7.75	48.58	80	[39]	6	0	0	0	this work^39^	[39]
Andalucía	AND	NA	−6	37.5	100	[16]	47	0	0	0	this work^16^	NA
Aragón	ARA	Aragón	−0.86	41.63	92	[17]	34	0	0	0	[17]	[26]
Asturias	AST	N. Central Spain	−5.87	43.34	63	[16]	27	0	0	0	[17]	[26]
Auvergne	AUV	France	3.09	45.78	89	[39]	5	0	0	0	this work^39^	[39]
Barcelona	BCN	Catalonia	2.13	41.4	571	(99)^16^, (472)^27^	245	11	8	6	this work^16, 27^	[26], [27]
Brittany	BRI	NA	−4.49	48.39	145	[16]	28	0	0	0	this work^16^	NA
Cantabria	CAN	N. Central Spain	−3.83	43.35	96	[16]	43	0	0	0	[16], [17]	[41]
Castelló	CAS	Valencia	−0.06	40	49	[27]	22	2	0	1	this work^27^	[27]
Galicia	GAL	NA	−8.55	42.88	70	[16]	28	0	0	0	this work^16^	NA
Girona	GIR	Catalonia	2.81	41.99	131	[27]	42	2	3	0	this work^27^	^27^
Île-de-France	IDF	France	2.35	48.86	91	[39]	9	2	0	0	this work^39^	^39^
Ireland	IRE	NA	−8	53	146	[16]	1	0	0	0	this work^16^	NA
Lleida	LLE	Catalonia	0.62	41.62	104	[27]	52	2	0	0	this work^27^	^27^
Madrid	MAD	NA	−3.72	40.42	99	[16]	49	0	0	0	this work^16^	NA
Mallorca	MAL	Mallorca	3	39.63	48	[27]	24	0	2	0	this work^27^	[27]
Midi-Pyrénées	MPY	France	1.45	43.61	67	[39]	7	1	0	0	this work^39^	[39]
Native Basque	NBA	Basques	−2.47	43.17	229	[16]	169	0	0	0	^16, 17^	[16]
Nord-Pas-de-Calais	NPC	France	3.04	50.63	68	[39]	8	3	0	0	this work^39^	[39]
Portugal	POR	NA	−8.5	39.5	109	[16]	44	0	0	0	this work^16^	NA
Provence–Alpes-Côte d’Azur	PAC	France	5.45	43.29	45	[39]	5	3	0	0	this work^39^	[39]
Pyrenees	PYR	Catalonia	1.12	42.49	46	[27]	24	0	1	0	this work^27^	[27]
Resident Basque	RBA	N. Central Spain	−3.69	42.35	111	[16]	53	0	0	0	[16], [17]	[16]
Tarragona	TAR	Catalonia	1.17	41.12	120	[27]	47	0	1	1	this work^27^	[27]
Valencia	VAL	Valencia	−0.4	39.48	79	[27]	31	3	2	1	this work^27^	[27]
Total					2990		1072	29	17	9		

Region: higher population grouping used in some analyses. (1): Chromosomes predicted to be R1b but without further SNP typing; (2): Chromosomes known to be R1b-P312* (xZ195, U152, L21) but without further typing; (3): Chromosomes known to be R1b-Z195 (xM167, Z220), but not typed for L176.

**Figure 3:**
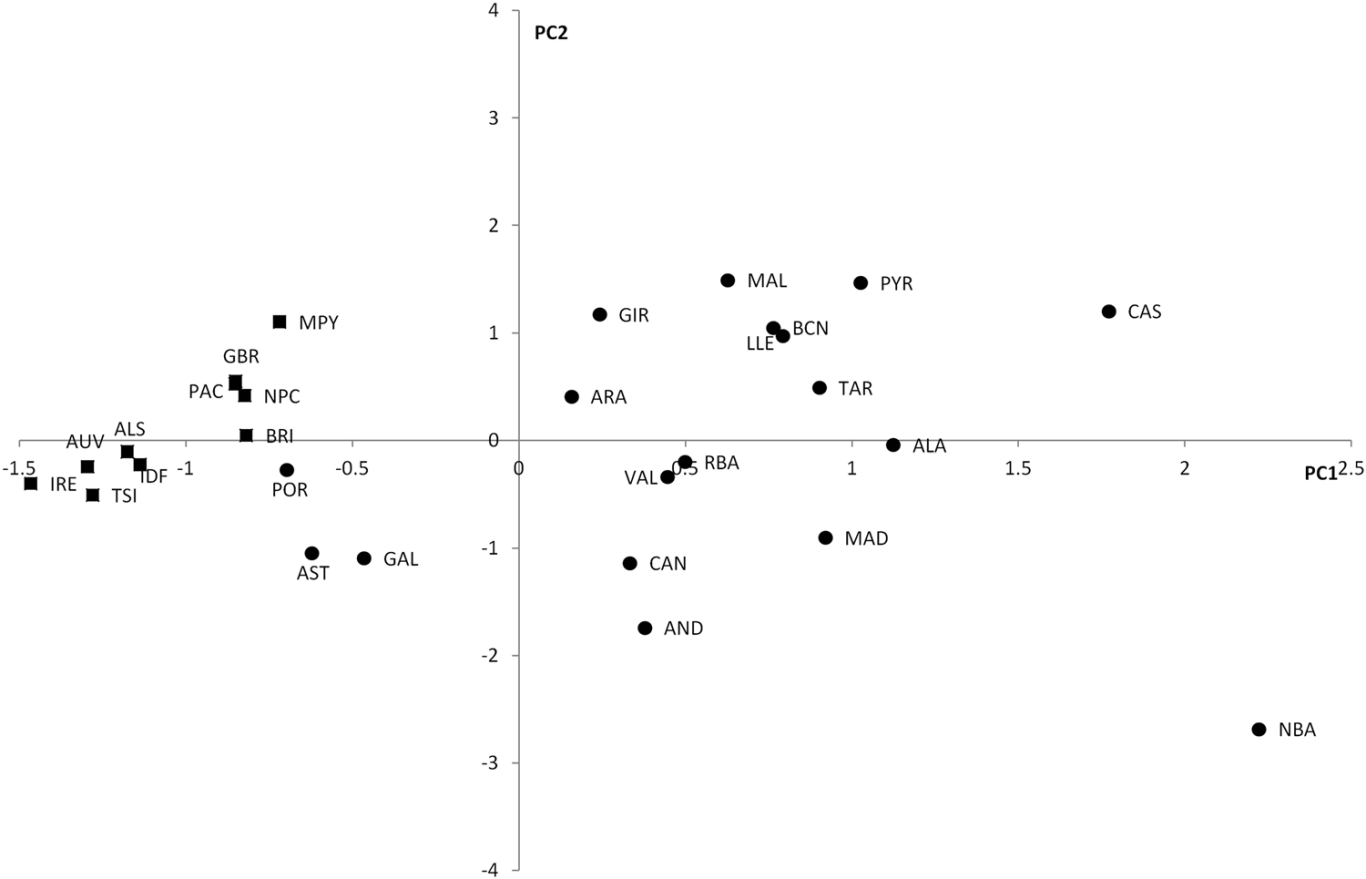
Principal component analysis of subhaplogroup frequencies. Population abbreviations as in Table 1. Circles: Iberian populations; squares: non-Iberian populations.

The subhaplogroup frequencies were summarized in a PC plot (Fig. 3). The first PC separated the Iberian populations (save for the three westernmost samples, namely Portugal, Galicia, and Asturias) from the rest, explained 68.6% of the total variation, and was positively correlated with DF27 and all of its subhaplogroups. On the contrary, PC2 (20.9%) was positively correlated with L176.2 and M167 and most negatively correlated with Z278 and M153, and separated most Eastern Iberian populations from the rest.

In order to quantify the structure of subhaplogroup frequencies, we performed AMOVA with several population groupings. Thus, if we compared Iberian populations vs. the rest, the proportion of the variance explained by the differences among these two groups (i.e., *F*_*CT*_) was 12.40% (p < 10^−4^), while the proportion of the variance found within groups (i.e., *F*_*SC*_) was 3.20% (p < 10^−4^). If the native Basques were split from the Iberians, then *F*_*CT*_ = 13.57% (p < 10^−4^) and *F*_*SC*_ = 1.37% (p < 10^−4^). Finally, if Eastern Iberians are also split from the rest of Iberians, then *F*_*CT*_ = 11.68% (p < 10^−4^) and *F*_*SC*_ = 0.31% (p = 0.0106). In conclusion, the differences among the groups that are apparent in the PCA plot are highly statistically significant.

**Figure 4:**
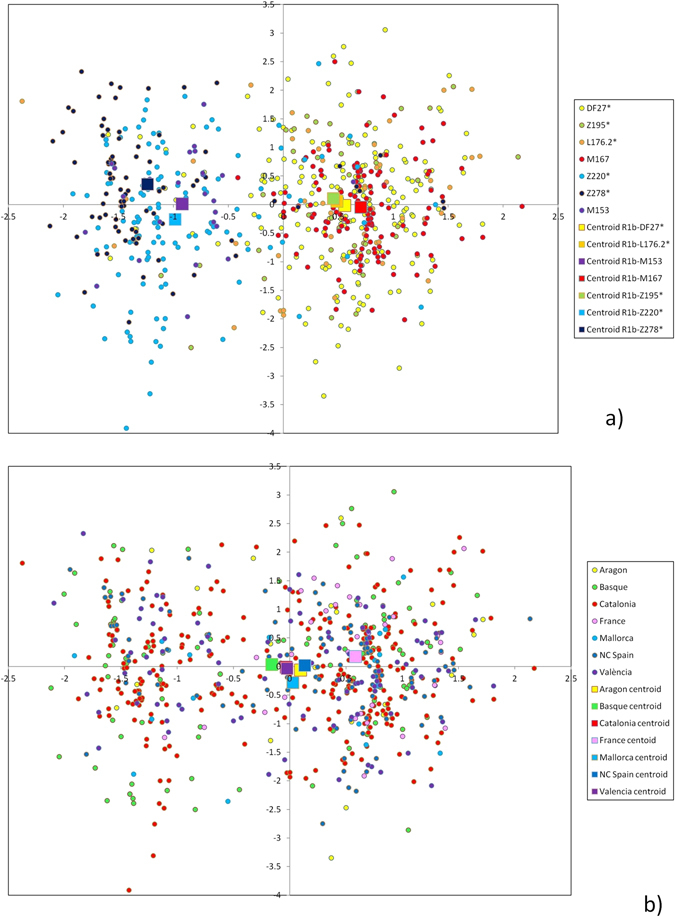
Principal component analysis of STR haplotypes. (**a**) Colored by subhaplogroup, (**b**) colored by population. Larger squares represent subhaplogroup or population centroids.

Haplotypes comprising 17 Y-STRs were available for 758 individuals (Table 2). AMOVA among this set of populations gave *R*_*ST*_ = 0.72% (p = 0.00386), while, for the same populations, subhaplogroup frequencies yielded *F*_*ST*_ = 8.33% (p < 10^−4^). Thus, Y-STRs seem to capture much less phylogeographic structure than SNPs themselves, as described for R1b-M269^11, 12^. Still, some Y-STR structure may be present within R1b-DF27^12, 17^. Since a median-joining tree with 688 different haplotypes is unmanageable, we resorted to principal component analysis (PCA) among haplotypes (Fig. 4). The first PC explained 15.1% of the STR variation and correlated mostly with DYS437 (r = 0.865), DYS448 (r = 0.858), and YGATAH4 (r = 0.724), and separated haplotypes that carried the derived allele for Z220 (that is, belonging to R1b-Z220*, R1b-Z278* and R1b-M153) from the rest. PC1 coordinates were highly significantly different by subhaplogroup (p ~ 10^−150^, ANOVA). The median haplotype for Z220-derived chromosomes was 11-14-18 at YGATAH4-DYS437-DYS448 while it was 12-15-19 for the rest of DF27 chromosomes (other STRs showed the same median allele). PC2 explained 8.6% of the STR variation and correlated with DYS390 (r = 0.647) and DYS456 (r = −0.544), and separated R1b-Z220* from R1b-Z278* chromosomes; overall, R1b-DF27 subhaplogroups had significantly different PC2 coordinates (p = 9.12 × 10^−4^). The same PC results can also be analyzed by population (Fig. 4b): PC1 coordinates are statistically significantly different by population (ANOVA, p = 0.00512), with the French samples having higher, positive values in this PC, while PC2 is not significant across populations (p = 0.781). These results can be explained by the very low frequency of Z220-derived chromosomes outside of Iberia.

**Table 2:** Diversity parameters for STR variation within R1b-DF27 chromosomes. K: number of different haplotypes; Dhap: haplotype diversity; Var: average STR allele repeat size variance; sd: standard deviation across loci of Var.

Population	N	K	Dhap	Var	sd (Var)
All	758	688	0.9996	0.330	0.215
Alacant	57	56	0.9994	0.377	0.400
Alsace	5	5	1	0.260	0.292
Aragón	29	29	1	0.372	0.218
Asturias	26	26	1	0.283	0.274
Auvergne	5	5	1	0.187	0.125
Native Basques	154	122	0.9951	0.282	0.174
Barcelona	184	178	0.9995	0.348	0.226
Cantabria	30	30	1	0.403	0.364
Castelló	23	23	1	0.294	0.213
Girona	33	33	1	0.306	0.183
Île-de-France	8	8	1	0.323	0.204
Lleida	39	39	1	0.314	0.187
Mallorca	21	21	1	0.341	0.280
Midi-Pyrénées	7	7	1	0.308	0.314
Nord-Pas-de-Calais	7	7	1	0.432	0.459
Provence–Alpes-Côte d’Azur	3	3	1	0.156	0.278
Pyrenees	17	17	1	0.443	0.328
Resident Basques	49	49	1	0.285	0.188
Tarragona	38	38	1	0.351	0.276
Valencia	23	23	1	0.337	0.152

### Internal diversity and ages of DF27 and its derived subhaplogroups

**Figure 5:**
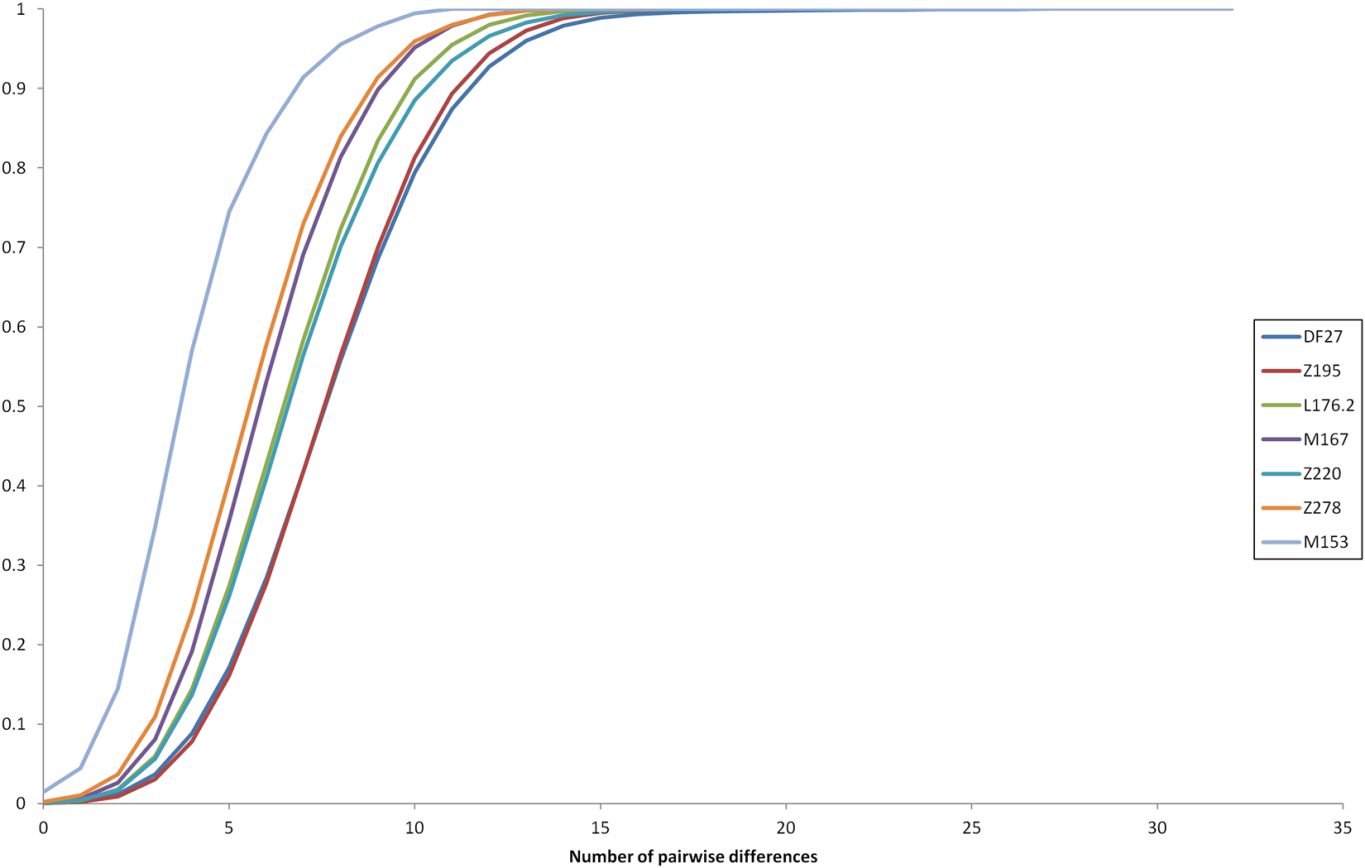
Cumulative distributions of the number of pairwise absolute differences in repeat size among individuals, by subhaplogroup.

The average STR variance of DF27 and each subhaplogroup is presented in Suppl. Table 2. As expected, internal diversity was higher in the deeper, older branches of the phylogeny. If the same diversity was divided by population, the most salient finding is that native Basques (Table 2) have a lower diversity than other populations, which contrasts with the fact that DF27 is notably more frequent in Basques than elsewhere in Iberia (Suppl. Table 1). Diversity can also be measured as pairwise differences distributions (Fig. 5). The distribution of mean pairwise differences within Z195 sits practically on top of that of DF27; L176.2 and Z220 have similar distributions, as M167 and Z278 have as well; finally, M153 shows the lowest pairwise distribution values. This pattern is likely to reflect the respective ages of the haplogroups, which we have estimated by a modified, weighted version of the ρ statistic (see Methods).

We estimated an age of 4190 ± 140 ya for the whole of DF27. This figure is remarkably similar both to the estimate (4128 ± 71 ya) that can be produced from whole Y-chromosome sequence variability in the 88 DF27-derived individuals present overall in the 1000 genomes project dataset, and to the age estimated from 201 individuals in our dataset for which 21 non-duplicated Y-STRs from the Powerplex Y23 System were available^26^ (3880 ± 165).

Z195 seems to have appeared almost simultaneously within DF27, since its estimated age is actually older (4570 ± 140 ya). Of the two branches stemming from Z195, L176.2 seems to be slightly younger than Z220 (2960 ± 230 ya vs. 3320 ± 200 ya), although the confidence intervals slightly overlap. M167 is clearly younger, at 2600 ± 250 ya, a similar age to that of Z278 (2740 ± 270 ya). Finally, M153 is estimated to have appeared just 1930 ± 470 ya.

Haplogroup ages can also be estimated within each population, although they should be interpreted with caution (see Discussion). For the whole of DF27, (Table 3), the highest estimate was in Aragon (4530 ± 700 ya), and the lowest in France (3430 ± 520 ya); it was 3930 ± 310 ya in Basques. Z195 was apparently oldest in Catalonia (4580 ± 240 ya), and with France (3450 ± 269 ya) and the Basques (3260 ± 198 ya) having lower estimates. On the contrary, in the Z220 branch, the oldest estimates appear in North-Central Spain (3720 ± 313 ya for Z220, 3420 ± 349 ya for Z278). The Basques always produce lower estimates, even for M153, which is almost absent elsewhere.

**Table 3:** Haplogroup ages estimated from STR variation with the weighted ρ method. 95% CI: 95% confidence interval.

Haplogroup ages		N	age	95% CI
DF27	total	758	4194	(4055, 4333)
	Aragón	29	4527	(3831, 5223)
	Basques	154	3931	(3617, 4245)
	Catalonia	311	4367	(4163, 4571)
	France	35	3428	(2911, 3945)
	Mallorca	21	4091	(3360, 4822)
	N. Central Spain	105	3614	(3293, 3935)
	Valencia	103	4525	(4166, 4884)
Z195	total	510	4569	(4398, 4740)
	Aragón	18	4177	(3293, 5061)
	Basques	83	3257	(2869, 3645)
	Catalonia	246	4584	(4349, 4819)
	France	34	3450	(2923, 3977)
	Mallorca	14	4028	(3081, 4975)
	N. Central Spain	44	3917	(3407, 4427)
	Valencia	71	4541	(4114, 4968)
L176.2	total	189	2964	(2737, 3191)
	Aragón	10	2759	(1626, 3892)
	Basques	14	3059	(2222, 3896)
	Catalonia	108	2931	(2651, 3211)
	France	15	3016	(2273, 3759)
	Mallorca	7	1465	(440, 2490)
	N. Central Spain	8	2365	(1465, 3265)
	Valencia	27	2933	(2370, 3496)
M167	total	137	2602	(2351, 2853)
	Aragón	4	3458	(1919, 4997)
	Basques	7	1221	(217, 2225)
	Catalonia	81	2597	(2289, 2905)
	France	12	2812	(2010, 3614)
	Mallorca	6	1626	(466, 2786)
	N. Central Spain	6	2050	(1082, 3018)
	Valencia	21	2404	(1818, 2990)
Z220	total	267	3318	(3114, 3522)
	Aragón	7	1956	(1080, 2832)
	Basques	62	2693	(2291, 3095)
	Catalonia	123	3380	(3094, 3666)
	Mallorca	7	3198	(2079, 4317)
	N. Central Spain	27	3718	(3105, 4331)
	Valencia	40	3683	(3166, 4200)
Z278	total	130	2745	(2475, 3015)
	Aragón	3	1716	(464, 2968)
	Basques	46	2298	(1877, 2719)
	Catalonia	41	2425	(1994, 2856)
	N. Central Spain	20	3417	(2733, 4101)
	Valencia	20	3084	(2369, 3799)
M153	total	34	1926	(1454, 2398)
	Basques	15	1582	(933, 2231)
	Catalonia	9	1202	(438, 1966)
	Valencia	8	2504	(1393, 3615)

### R1b-DF27 and demography

We tested the dynamics of R1b-DF27 by means of Approximate Bayesian Computing (ABC). In particular, we compared two simple models: constant population size vs. growth since time Tstart. Both the rejection and the regression method undoubtedly favoured the growth model, with associated posterior probabilities that were never lower than 0.99. The principal component analysis (PCA) of the first 3,000 best simulations from each model (i.e. the 3,000 simulation closest to the observed dataset that are generated by each model) actually shows that the point corresponding to the observed data falls in the middle of the results obtained simulating growth, thus confirming that the growth model is also able to generate the observed variation (Suppl. Fig. 2). We then estimated the demographic parameters associated with the growth model. The median value for Tstart has been estimated at 103 generations (Table 4), with a 95% highest probability density (HPD) range of 50–287 generations; effective population size increased from 131 (95% HPD: 100–370) to 72,811 (95% HPD: 52,522–95,334). Considering patrilineal generation times of 30–35 years^27^, our results indicate that R1b-DF27 started its expansion ~3,000–3,500 ya, shortly after its TMRCA.

**Table 4:** ABC results for R1b-DF27 and reference haplogroups. GSM: Generalized Stepwise Mutation; HPD: Highest Probability Density.

R1b-DF27	Mean	Median	Mode	95% HPD-LowB	95% HPD-UppB
Tstart	128	103	75	50	287
Na	162	131	100	100	350
Nc	72685	72812	73624	52523	95334
GSM	0.047	0.046	0.044	0.004	0.089
R1b-S116					
Tstart	50	50	50	50	52
Na	182	134	110	100	370
Nc	99844	99854	99860	99690	100000
GSM	0.239	0.240	0.246	0.094	0.380
G2a					
Tstart	82	61	51	50	175
Na	145	122	100	100	340
Nc	10448	7984	5865	1549	25267
GSM	0.179	0.177	0.179	0.023	0.325
I2					
Tstart	82	67	58	50	136
Na	178	140	100	100	370
Nc	40622	37366	28135	9162	81946
GSM	0.036	0.027	0	0	0.099
J2a					
Tstart	55	52	51	50	61
Na	154	129	100	100	350
Nc	94264	95421	96953	85633	100000
GSM	0.022	0.017	0.000	0.000	0.069

As a reference, we applied the same analysis to the whole of R1b-S116, as well as to other common haplogroups such as G2a, I2, and J2a. Interestingly, all four haplogroups showed clear evidence of an expansion (p > 0.99 in all cases), all of them starting at the same time, ~50 generations ago (Table 4), and with similar estimated initial and final populations. Thus, these four haplogroups point to a common population expansion, even though I2 (TMRCA, weighted ρ, 7,800 ya) and J2a (TMRCA, 5,500 ya) are older than R1b-DF27. It is worth noting that the expansion of these haplogroups happened after the TMRCA of R1b-DF27.

## Discussion

We have characterized the geographical distribution and phylogenetic structure of haplogroup R1b-DF27 in W. Europe, particularly in Iberia, where it reaches its highest frequencies (40–70%). The age of this haplogroup appears clear: with independent samples (our samples vs. the 1000 genome project dataset) and independent methods (variation in 15 STRs vs. whole Y-chromosome sequences), the age of R1b-DF27 is firmly grounded around 4000–4500 ya, which coincides with the population upheaval in W. Europe at the transition between the Neolithic and the Bronze Age^2, 9^. Before this period, R1b-M269 was rare in the ancient DNA record, and during it the current frequencies were rapidly reached^2, 9, 10^. It is also one of the haplogroups (along with its daughter clades, R1b-U106 and R1b-S116) with a sequence structure that shows signs of a population explosion or burst^1^. STR diversity in our dataset is much more compatible with population growth than with stationarity, as shown by the ABC results, but, contrary to other haplogroups such as the whole of R1b-S116, G2a, I2 or J2a, the start of this growth is closer to the TMRCA of the haplogroup. Although the median time for the start of the expansion is older in R1b-DF27 than in other haplogroups, and could suggest the action of a different demographic process, all HPD intervals broadly overlap, and thus, a common demographic history may have affected the whole of the Y chromosome diversity in Iberia. The HPD intervals encompass a broad timeframe, and could reflect the post-Neolithic population expansions from the Bronze Age to the Roman Empire^28^.

While when R1b-DF27 appeared seems clear, where it originated may be more difficult to pinpoint. If we extrapolated directly from haplogroup frequencies, then R1b-DF27 would have originated in the Basque Country; however, for R1b-DF27 and most of its subhaplogroups, internal diversity measures and age estimates are lower in Basques than in any other population. Then, the high frequencies of R1b-DF27 among Basques could be better explained by drift rather than by a local origin (except for the case of M153; see below), which could also have decreased the internal diversity of R1b-DF27 among Basques. An origin of R1b-DF27 outside the Iberian Peninsula could also be contemplated, and could mirror the external origin of R1b-M269, even if it reaches there its highest frequencies. However, the search for an external origin would be limited to France and Great Britain; R1b-DF27 seems to be rare or absent elsewhere: Y-STR data are available only for France, and point to a lower diversity and more recent ages than in Iberia (Table 3). Unlike in Basques, drift in a traditionally closed population seems an unlikely explanation for this pattern, and therefore, it does not seem probable that R1b-DF27 originated in France. Then, a local origin in Iberia seems the most plausible hypothesis. Within Iberia, Aragon shows the highest diversity and age estimates for R1b-DF27, Z195, and the L176.2 branch, although, given the small sample size, any conclusion should be taken cautiously. On the contrary, Z220 and Z278 are estimated to be older in North Central Spain (N Castile, Cantabria and Asturias). Finally, M153 is almost restricted to the Basque Country: it is rarely present at frequencies >1% elsewhere in Spain (although see the cases of Alacant, Andalusia and Madrid, Suppl. Table 1), and it was found at higher frequencies (10–17%) in several Basque regions^25^; a local origin seems plausible, but, given the scarcity of M153 chromosomes outside of the Basque Country, the diversity and age values cannot be compared.

Within its range, R1b-DF27 shows same geographical differentiation: Western Iberia (particularly, Asturias and Portugal), with low frequencies of R1b-Z195 derived chromosomes and relatively high values of R1b-DF27* (xZ195); North Central Spain is characterized by relatively high frequencies of the Z220 branch compared to the L176.2 branch; the latter is more abundant in Eastern Iberia. Taken together, these observations seem to match the East-West patterning that has occurred at least twice in the history of Iberia: i) in pre-Roman times, with Celtic-speaking peoples occupying the center and west of the Iberian Peninsula, while the non-Indoeuropean eponymous Iberians settled the Mediterranean coast and hinterland; and ii) in the Middle Ages, when Christian kingdoms in the North expanded gradually southwards and occupied territories held by Muslim fiefs.

### The relevance and possible applications of R1b-DF27

Although R1b-DF27 as a whole has remained relatively obscure in the academic literature, two of the SNPs it contains, namely M167 (SRY2627) and M153 have accrued quite a number of studies. Thus, excluding this paper, M153 has been typed in 42 populations, for a total of 3,117 samples^22, 23, 25, 29, 30^; M167 has been typed in at least 113 populations and 10,379 individuals^4, 18–20, 22–25, 29–31^. It is not obvious then why both markers are absent from Y-phylotree (http://www.phylotree.org/Y/tree/index.htm, ref. 32), which is the current academic Y-chromosome haplogroup reference tree and which contains within DF27 a number of much more obscure SNPs.

Potentially, a SNP with relatively high frequencies in Iberian and Iberian-derived populations and rarer elsewhere could be applied in a forensic genetics setting to infer the biogeographic origin of an unknown contributor to a crime scene^33^. However, neither the specificity nor the sensitivity of such an application would guarantee significant investigative leads in most cases. When compared to the 1000 genomes CEU sample of European-Americans^15^, R1b-DF27 is just 4.19 times more frequent in Iberians than in CEU, a ratio that raises to 6.82 for R1b-Z220 (which, though, has a frequency of only 13.9% in Iberians). Probably, other types of evidence of the involvement of a person of interest of Iberian descent would be needed to justify tying R1b-DF27.

R1b-DF27 may also be used to trace migratory events involving Spanish or Portuguese men, particularly outside of Western Europe; a clear example can be seen the Latin American populations (see the Introduction section), where R1b-DF27 seems to correlate with the amount of male-mediated Spanish admixture: it is clearly less frequent in the populations with a stronger Native American component, such as Mexico and Peru. Even within Europe, Y haplogroup frequencies have been used to detect short-range migration events, such as that from Northern France to Flanders^34^. Thus, the traces of the medieval expansion of the Aragon kingdom towards the Mediterranean in the 14th–15th centuries, or the Castilian occupation of Flanders in the 17th century may be traced through the male lineages, R1b-DF27 in particular.

Finally, the Y chromosome in often studied in connection with surnames, since the latter are also often transmitted through the male line^35^. For that, Y-STR haplotypes are analyzed, and, given the Y-STR mutation rates, similarity in Y-STR haplotypes between men sharing the same surname is taken as indicative of a shared genealogical origin^36, 37^. However, diversity in Y-STR haplotypes within the R1b-M269 branch is rather small^11, 12^, and the sole use of Y-STRs may result in homoplasy, rather than shred origin, causing Y-STR haplotype convergence. Thus, particularly within Iberia, R1b-DF27 should be used when trying to ascertain the founding events of surnames. No SNP deeper than R1b-M269 was typed in a survey of Spanish surnames^38^, while some SNPs in the R1b-DF27 branch (Z195, Z220, Z278, M153 and M167) were used in a similar study^27^.

Although we have contributed to the understanding of the phylogeography of R1b-DF27, which makes up a dominant fraction of Iberian (and Latin American) Y chromosomes, better tools and designs would be needed to solve some of the issues we discussed above. In particular, we genotyped pre-ascertained SNPs, and a global characterization of the whole sequence diversity of this haplogroup would allow more precise statistical analyses to be run. Also, a more comprehensive sampling scheme, including more information from Atlantic Iberia, would be desirable to obtain a more accurate picture of this haplogroup.

## Methods

### Samples/ethics

The population samples we analyzed comprised a total of 2990 individuals, of which 1072 carried the derived allele at the DF27 SNP. Additionally, 55 individuals with partial information were used to estimate subhaplogroup frequencies (see below). These samples cover the Iberian Peninsula and France, and were originally described in^16, 27, 39^ (Table 1, Fig. 2). Also, subhaplogroup frequencies were estimated for the British (GBR) and Tuscan (TSI) samples of the 1000 genomes project^15^. Informed consent for study participation was obtained from all the subjects; Internal Review Board approval for this work was granted by Faculty of Pharmacy UPV/EHU, September 26th 2008; CEISH/119/2012, BNADN Ref. 12/0031; and CEIC-PSMAR ref. 2016/6723/I. This research was conducted under the principles of the Helsinki declaration.

### SNP genotyping

All samples were typed for SNPs/indels M269, S116 (P312), DF27, Z195, L176, M167 (SRY2627), Z220 (S356), Z278, and M153 (Fig. 1). DF27, Z195, L176, M167, Z220, Z278, and M153 were typed in samples from Portugal, Andalusia, Galicia, Madrid, and part of the Alacant and Barcelona samples as described in ref. 17. The original genotyping of the French samples (except Brittany)^39^ was supplemented with the SNPs in the Open Array panel described in ref. 27. Subsequently, the French samples plus others from Eastern Iberia (see Table 1) were genotyped for DF27 and L176 by Sanger sequencing, since these polymorphisms were not part of the original Open Array panel. Both were amplified using 2.5 μl buffer, 2 μl dNTPs, 1.25 μl each of forward/reverse primers, 1.5 μl MgCl_2_, 0.2 μl Taq polymerase, 1.5 μl DNA, and 14.8 μl H_2_O. DF27 was first amplified with a nested PCR to reduce non-specific amplifications. The nested PCR involves two sets of primers used in two successive PCR amplifications, namely outer DF27 forward: GGGAATTTGATCCTGTCGTTG, outer DF27 reverse: GAACAAAGCCTCCAAGAAATATGAGG, M13F-tagged nested DF27 forward: TGTAAAACGACGGCCAGTTATTTTATTTCTCCTTCACTTATA, nested DF27 Reverse: ATCCAGGAGAACTTCCCCAATC. In the first PCR, 30 cycles were performed at 95 °C (30 sec), 60.5 °C (30 sec), and 72 °C (40 sec); in the second PCR, the annealing temperature was lowered to 59.2 °C. For L176, primers were L176 Forward: CAACAGGCCAGAAGGAACAG and L176 reverse: TTACAGGTGGAATGGGGTGT; the annealing temperature was 58.3 °C, and times and number of cycles were the same as in DF27.

Genotypes for 17 short tandem repeats (STRs) contained in the AmpFlSTR®YFiler® PCR Amplification kit (Applied Biosystems) were available for most populations (see Table 1)^16, 26, 27, 39–41^. The dataset generated during the current study is available from the corresponding author on reasonable request.

### Statistics

For most populations, the frequencies of DF27 and its subhaplogroups were estimated by direct counting. However, in some populations, individuals with partial information were present: in some cases, no SNP information was available, but they could be inferred to carry R1b from their STR haplotypes^42, 43^; further subhaplogroup inference is precluded by the high homogeneity of STR haplotypes within R1b-M269^11, 12^. In other cases, individuals were known to be S116 (xZ195, L21, U152) or Z195 (xM167, Z220), but further genotyping for DF27 or L176 failed. The relative proportions of cases with full genotypes over R1b, S116 or Z195 were used to estimate the probabilities of each individual with missing genotypes to belong to each possible subhaplogroup. Using these probabilities as frequencies, the frequency of each subhaplogroup was estimated. Detailed formulas for each subhaplogroup are given in Supplementary note 1. Individuals with missing information were used only to refine the estimation of subhaplogroup frequencies.

Haplogroup frequency maps were drawn with SURFER v. 12 (Golden Software, Golden CO, USA) by krigging. Principal component analysis was performed with IBM SPSS Statistics v. 19. Basic descriptive statistics, as well as AMOVA, were computed with Arlequin 3.5^44^. Haplogroups were dated from STR variation with ρ_w_, a weighted version of ρ^45^ that leverages on the relatively precise knowledge of the mutation rate of each Y-STR. Thus, it takes into account that mutations at slow STRs take longer to accumulate than mutations at faster STRs. It is defined as

$${\rho }_{W}=\frac{1}{N}\sum _{i=1}^{k}{n}_{i}(\sum _{j=1}^{S}(|{X}_{ji}-{X}_{jm}|)\cdot \frac{\bar{\mu }}{{\mu }_{j}})$$

where *N* is the number of chromosomes, *k* is the number of different haplotypes, *n*_*i*_ is the absolute frequency of the *ith* haplotype, *S* is the number of different STRs, *X*_*ji*_ is the allelic state of the *ith* haplotype at the *jth* STR, *X*_*jm*_ is the median allele at the *jth* STR, $\bar{\mu }$ is the average mutation rate and μ_*j*_ is the mutation rate of the *jth* STR. The standard deviation of *ρ*_*W*_ is given by

1 $$sd({\rho }_{W})=\frac{1}{N}\sqrt{\sum _{i=1}^{k}{n}_{i}^{2}(\sum _{j=1}^{S}(|{X}_{ji}-{X}_{jm}|)\cdot \frac{\bar{\mu }}{{\mu }_{j}})}$$

And age, as in ref. 45, is estimated as

2 $$T={\rho }_{W}\cdot \bar{\mu }$$

where $\bar{\mu }$ is now expressed in years per mutation. *ρ*_*W*_ was computed with an *ad hoc* R script, which is available in github (http://github.com/fcalafell/weighted_rho). Mutation rates were retrieved from the Y-Chromosome STR Haplotype Database (YHRD, www.yhrd.org) on Feb. 1, 2017. DYS385 was omitted from the calculations, and DYS389I was subtracted from DYS389II. Additionally, outlier individuals were detected and removed from the estimate as suggested in ref. 20.

Unweighted ρ was used to estimate the age of DF27 by using the whole Y chromosome sequences of the 88 unrelated individuals derived for this SNP and present in the 1000 genomes project dataset. The mutation rate considered was 0.888 × 10^−9^ per year^1, 46^, which, taking into account the ~10.36 Mb of the Y chromosome amenable to short-read sequencing and SNP detection^1^, translates to a rate of 108.7 years/mutation.

Approximate Bayesian Computing (ABC) was used to test alternative demographic models and to estimate their parameters. One million simulations were run with *fastsimcoal2* ^47, 48^, either with a constant population size (drawn from a lognormal distribution between 100 and 100,000), or with an exponential growth that started Tstart generation ago. In the growth model, the effective population sizes before (Na) and at the end (Nc) of the growth were drawn in the same fashion of the constant model, and conditioned to Na < Nc. Na refers to a time Tstart drawn from a uniform distribution between 50 and 350 generations. STR mutation rates were taken as fixed given the high precision with which they are known, but the value of the geometric parameter for the Generalized Stepwise Mutation model was sampled from a uniform distribution with limits (0; 0.8). To summarize the data, we calculated the mean and the standard deviation over loci of four statistics: the number of different haplotypes (K), the haplotype diversity (H), the allelic range and the Garza- Williamson’s index. We calculated posterior probabilities of the models by means of the simple rejection algorithm^49^ as well as of the weighted multinomial logistic regression^50^, evaluating different thresholds for both methods to check the stability of the results as in ref. 51. For parameter estimation, we calculated the Euclidian distance between the simulated and observed summary statistics and retained the 5% of the total simulations corresponding to the shortest distances. Posterior probability for each parameter was estimated using a weighted local regression^52^, after a logtan transformation^53^.

## Electronic supplementary material

Supplementary materials

Supplementary Dataset 1

Supplementary Dataset 2
