# Supplementary material for: Analysis of the R1b-DF27 haplogroup shows that a large fraction of Iberian Y-chromosome lineages originated recently in situ
Source: Sci Rep. 2017 Aug 4;7:7341. doi: 10.1038/s41598-017-07710-x (PMC5544771; doi:10.1038/s41598-017-07710-x)
Supplement: Supplementary file 1 — Supplementary materials [file 41598_2017_7710_MOESM1_ESM.pdf]

Analysis of the R1b-DF27 haplogroup shows that a large fraction of Iberian Y-chromosome lineages originated recently in situ.

Neus Solé-Morata, Patricia Villaescusa, Carla García-Fernández, Neus Font-Porterias, María José Illescas, Laura Valverde, Francesca Tassi, Silvia Ghirotto, Claude Férec, Karen Rouault, Susana Jiménez-Moreno, Begoña Martínez-Jarreta, Maria Fátima Pinheiro, María T. Zarrabeitia, Ángel Carracedo, Marian M. de Pancorbo, Francesc Calafell.

Supplementary materials:

Supplementary note.

Supplementary Figure 1. R1b-DF27 in the context of the Y-SNP tree compiled in ref. [32] and available from http://www.phylotree.org/Y/tree/index.htm. In red, SNPs typed in this work. In parentheses, SNPs absent from phylotree-Y. Comas separate different SNPs falling the same phylogenetic branch, while slashes indicate alternate names for the same SNP.

Supplementary Figure 2. Additional frequency contour maps of paragroups, and of SRY2627 and of M153 with additional data from the literature. Maps were drawn with SURFER v. 12 (Golden Software, Golden CO, USA).

Supplementary Figure 3. Principal component analysis of summary statistics in stationary and growth ABC simulations; the observed value falls clearly within the cloud of growth simulations.

Supplementary note 1.

Let *a* be the absolute frequency of haplogroup R1b-M269(xP312) in a sample of *n* Y chromosomes; similarly, let *b*: R1b-P312(xDF27), *c*: R1b-DF27(xZ195), *d*: R1b-Z195(xL176,Z220), *e*: R1b-L176(xM176), *f*:R1b-M167, *g*: R1b-Z220(xM278), *h*: R1b-Z278(xM153), and *i*: R1b-M153. Let *s*=*a*+*b*+*c*+...+*i*. We have three types of samples with partial information: R1b-M269 without further subtyping (let its frequency be *j*), R1b-P312 (xU152, L21, Z195), but not typed for DF27 (call it *k*), and R1b-Z195(xZ220), not typed for L176 (*l*). *j* individuals may belong to any of the *a*,...,*i* subhaplogroups with probability *a/s*,...,*i/s*; *k* can be R1b-DF27(xZ195) with probability *c/(b+c)*, and R1b-Z195(xZ220,M167) can be either R1b-Z195(xL176,Z220) with probability *d/(d+e)* or R1b-L176(xM167) with probability *e/(d+e)*. Combining these probabilities and turning them into estimated relative frequencies (which we denote with a circumflex over each letter), we have

$$\hat{c} = \frac{c\left(1 + \frac{j}{s} + \frac{k}{b+c}\right)}{n}$$

$$\hat{d} = \frac{d\left(1 + \frac{j}{s} + \frac{l}{d+e}\right)}{n}$$

$$\hat{e} = \frac{e\left(1 + \frac{j}{s} + \frac{l}{d+e}\right)}{n}$$

$$\hat{f} = \frac{f\left(1 + \frac{j}{s}\right)}{n}$$

$$\hat{g} = \frac{g\left(1 + \frac{j}{s}\right)}{n}$$

$$\hat{h} = \frac{h\left(1 + \frac{j}{s}\right)}{n}$$

$$\hat{\imath} = \frac{i\left(1 + \frac{j}{s}\right)}{n}$$

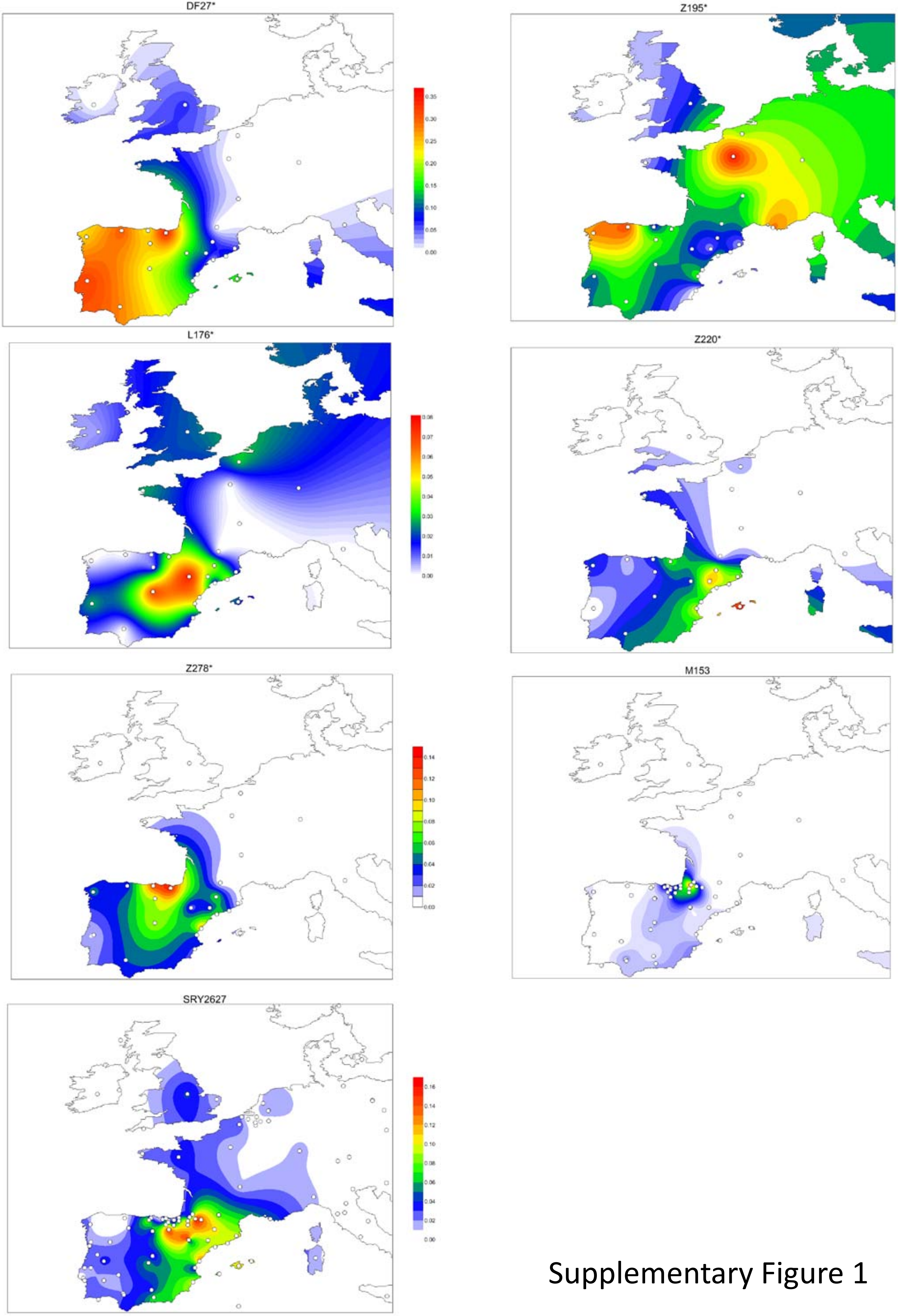

Supplementary Figure 1

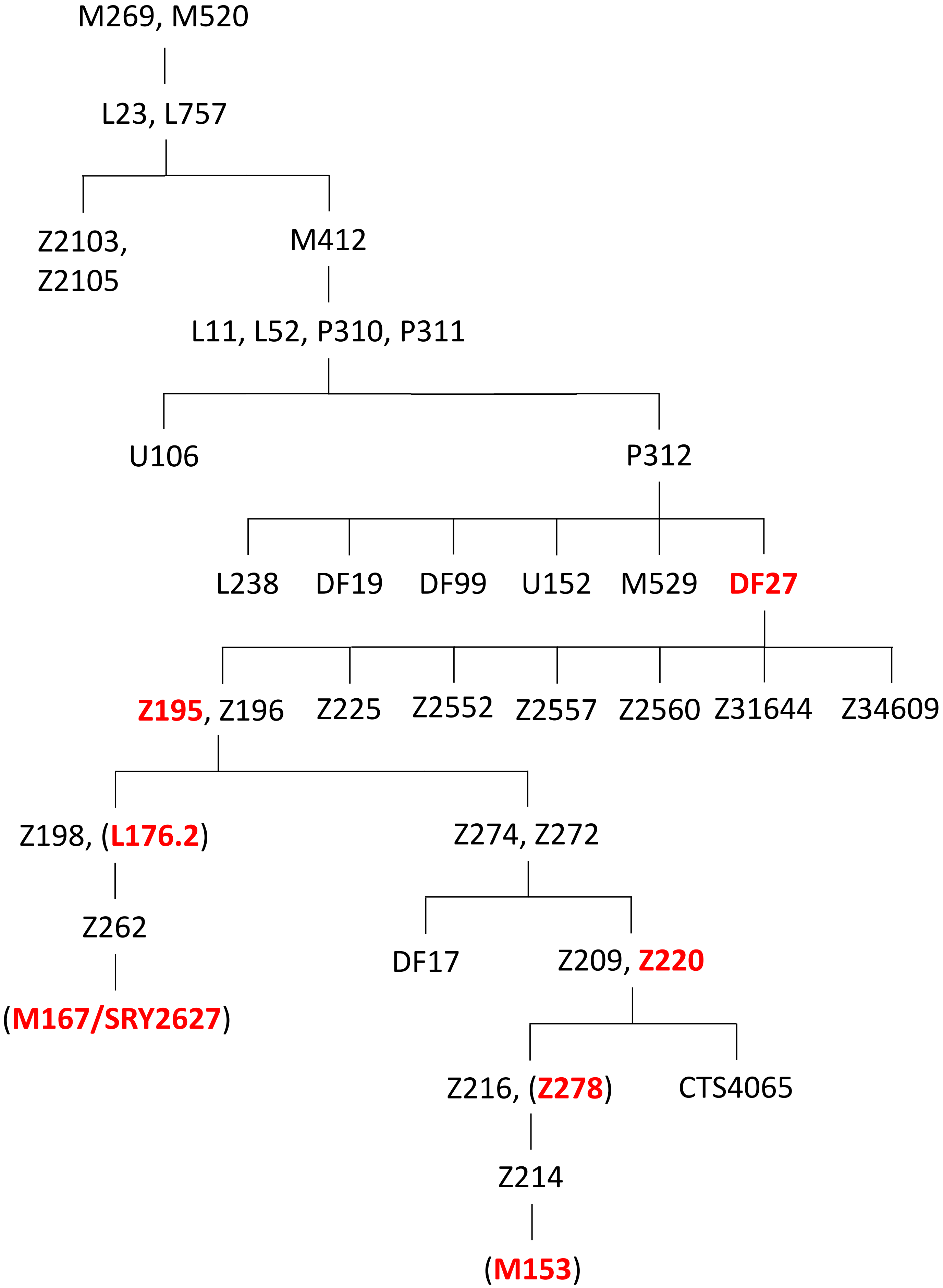
M269, M520
L23, L757
Z2103, Z2105
M412
L11, L52, P310, P311
U106
P312
L238
DF19
DF99
U152
M529
DF27
Z195, Z196
Z225
Z2552
Z2557
Z2560
Z31644
Z34609
Z198, (L176.2)
Z274, Z272
Z262
(M167/SRY2627)
DF17
Z209, Z220
Z216, (Z278)
CTS4065
Z214
(M153)

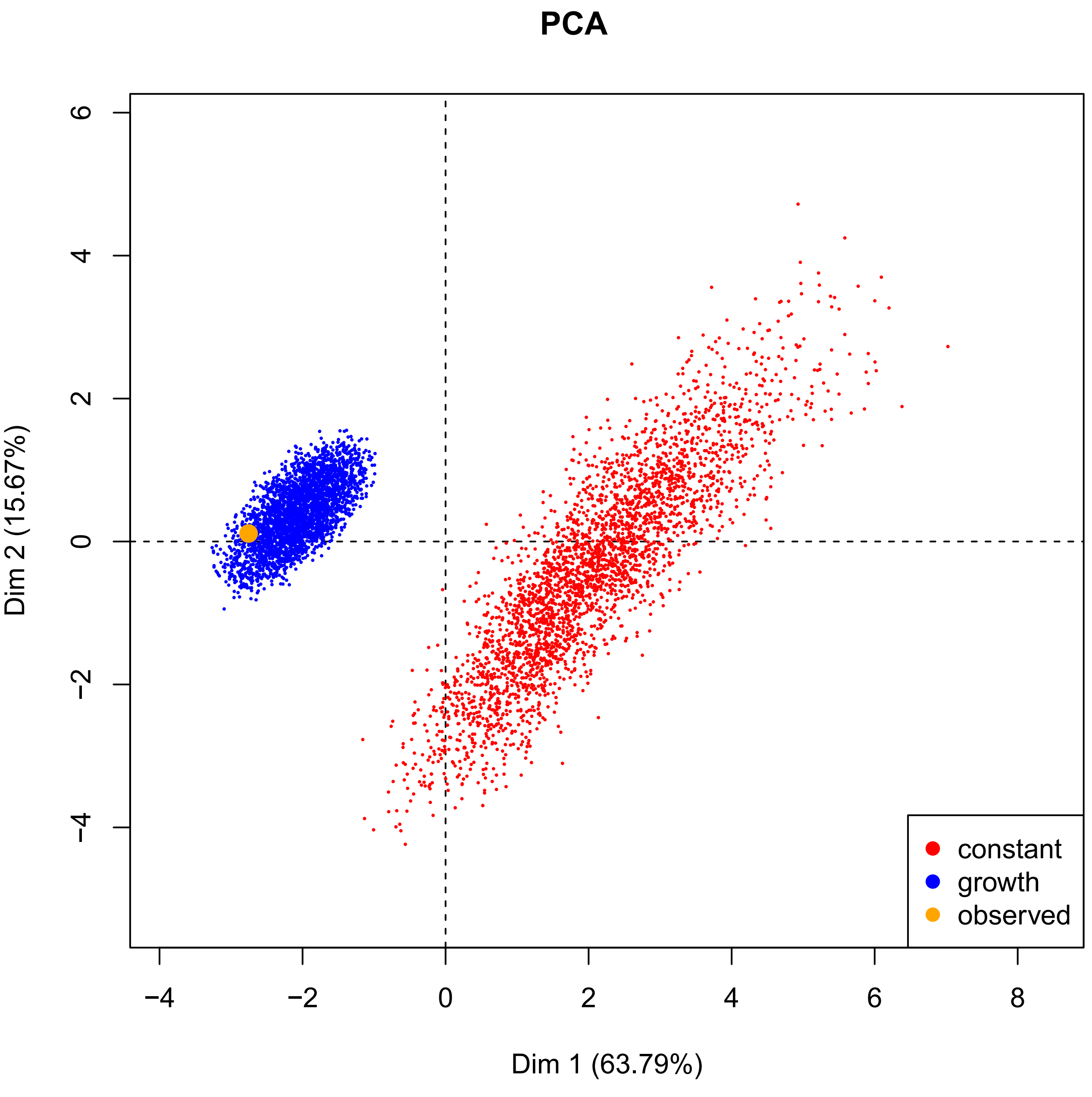
PCA
Dim 1 (63.79%)
Dim 2 (15.67%)
−4
−2
0
2
4
6
8
constant
growth
observed
